# Editorial: Environmental and endogenous signals: crop yield and quality regulation

**DOI:** 10.3389/fpls.2023.1271918

**Published:** 2023-08-21

**Authors:** Lei Gao, Yufeng Hu

**Affiliations:** State Key Laboratory of Crop Gene Exploration and Utilization in Southwest China, Sichuan Agricultural University, Chengdu, Sichuan, China

**Keywords:** crop yield and quality, source-sink-translocation, gene regulation, seed size, grain filling, storage component accumulation

Grain is the harvestable part of cereal crops, and the synthesis and accumulation of grain storage substances during the filling process is an important factor that determines cereal crop yield and quality. The process of source-sink-translocation in plants plays an essential role in determining grain filling. Studies have shown that grain filling is affected by changes in the expression of genes related to photosynthetic capacity, such as those associated with source-sink translocation (Brazel and Ó'Maoiléidigh, 2019; Sanchez-Bragado et al., 2020; Zhang et al., 2022). Additionally, nutritional transport systems such as transporters of nutrient elements (Shannon, 1972; Sosso et al., 2015; Bezrutczyk et al., 2018; Wang et al., 2018; Shen et al., 2022; Wang et al., 2022; Yang B. et al., 2022; Sun et al., 2023), plasmodesmata, and sieve plates are essential to grain filling determination (Zhang et al., 2010; You et al., 2021). Lastly, enzymes that synthesize storage starch (Hannah and James, 2008; Huang et al., 2021; Wang et al., 2021) and lipids (Yang Y. et al., 2022; Luo et al., 2023) and genes that encode storage proteins (Yang et al., 2023; Zhao et al., 2023) in seeds work together to determine grain yield and quality of the cereal crops. During these processes, various endogenous developmental and exogenous environmental cues including phytohormones such as abscisic acid (ABA) (Wang and Zhang, 2020; Wang et al., 2020), brassinosteroids (BR) (Wu et al., 2008; Song et al., 2023), and auxin (Balcerowicz, 2021; Zhao et al., 2022), signaling metabolites such as trehalose-6-phosphate (T6P) (Smeekens, 2015; Meitzel et al., 2021), and sucrose (Chen et al., 2019; Jiang et al., 2021), as well as environmental stressors (biotic and abiotic stresses) are rapidly integrated and translated into a variety of signals that regulate critical genes involved in grain development and filling. To develop high-yielding and high-quality cereal varieties, it is necessary to have a thorough understanding of the inherent regulatory mechanisms and genetic basis of grain filling and development.

Abiotic stress including cold temperature stress is one of the most important environmental factors affecting crop yield and quality. In a review article addressing this Research Topic, Hernández et al. presented an overview of the physiological and biochemical responses of sorghum to cold stress, by examining the molecular mechanisms through which sorghum copes with chilling stress via multiple pathways that are regulated by phytohormones, transcription factors, and microRNAs. The review explores how chilling stress impacts the photosynthetic efficiency of sorghum and consequently, its storage starch accumulation. PPDK, an enzyme essential to the photosynthetic pathway in C4 crops, becomes unstable, and ribulose-1,5-bisphosphate carboxylase/oxygenase (Rubisco) is inhibited under cold temperature stress. The consequence is a reduction in photosynthetic efficiency and a decrease in starch accumulation in grains, which in turn results in poor grain quality and yield.

About half of the population of the world is fed by rice, one of the most important food crops worldwide. As living standards improve around the world, the demand for high-quality rice is increasing. A variety of characteristics related to grain quality such as milling traits, physical appearance, cooking and taste properties, as well as nutritional quality and yield are often the focus of genetic research on rice. A high-resolution QTL mapping study was conducted by Jin et al. to investigate the milling quality and appearance quality of an F2 population derived from a cross between Koshihikari and Nona Bokra. Approximately 15 QTLs were identified for seven quality traits of rice grains comprising whole grain percentage, head rice percentage, head rice area percentage, transparency, chalky rice percentage, chalkiness area percentage, and chalkiness degree. Most of these QTL are novel to rice grain quality research. Several genes involved in starch synthesis and storage protein biosynthesis pathways were identified within these QTL regions. In a separate independent QTL mapping study, Mao et al. identified 13 QTLs associated with grain traits and yield in rice, such as grain length, grain width, and thousand-grain weights using a chromosomal segment substitution line of rice. In these QTL mapping studies, the pyramiding effect of some of the identified major QTLs was also estimated, providing novel and valuable genetic markers to improve rice grain quality and yield.

As the main storage component of cereal grains, starch plays an important role in determining grain yield and quality. Among the research articles published on this Research Topic, two deal with the regulation of starch accumulation in cereal grains. In one of the two articles, a protein, GF14f, an isoform of 14-3-3 proteins, was reported to negatively regulates carbon remobilization and starch accumulation in ratoon rice, thus reducing yield and causing grain chalkiness (Lin et al.). Inhibition of GF14f gene expression enhances the efficiency of translocation and assimilation of dry matter into grains and increases the gene expressions of key starch synthetic enzymes, AGPase, soluble starch synthase and starch branching enzymes during grain filling. By analyzing multiple transcriptomes of different tissues of sorghum at different periods, Xiao et al. dissect key genes associated with sorghum starch biosynthesis and potential regulatory transcription factors. This second research article explores the molecular mechanism by which NAC transcription factors participate in and regulate starch biosynthesis in sorghum.

Globally, heavy metal pollution has become one of the most important environmental concerns that causes fatal damage to cereal quality. In a genome-wide association study of 312 maize inbred lines under lead stress, Hou et al. identified four SNPs and candidate genes related to root bushiness. A combination of association analysis of candidate genes within significant SNP loci with transcriptomic data under lead stress reveals two variants in ZmbZIP107 that have significant association with root bushiness in governing lead-tolerance in maize. The role of ZmbZIP107 in controlling lead tolerance was validated in transgenic rice lines. In addition to providing a genetic basis for lead tolerance, their results contributed a novel gene for the development of lead-tolerant varieties of maize.

Overall, this Research Topic has been the subject of only five peer-reviewed original research articles and one review article, most of which deal with the regulation of cereal grain traits by environmental or genetic factors. These articles presented an overview of recent progress in the regulation of grain yield and quality traits and provided a useful reference for improving cereal yield and quality. Finally, we would like to express our gratitude to the authors, reviewers, and editors of this Research Topic. It is only through their efforts and dedication that this Research Topic was made possible.

## Author contributions

LG: Project administration, Writing – review & editing. YH: Project administration, Writing – original draft.
